# Design, Preparation, and Ex Vivo Skin Permeation of Doxepin Microemulsion System for Topical Delivery

**DOI:** 10.1111/jocd.16786

**Published:** 2025-01-21

**Authors:** Hossein Heidari Kaydan, Eskandar Moghimipour, Hossein Dalvand, Nasibeh Jamali, Arghavan Salimi, Anayatollah Salimi

**Affiliations:** ^1^ Department of Pharmaceutics, Faculty of Pharmacy Ahvaz Jundishapur University of Medical Sciences Ahvaz Iran; ^2^ Medicinal Plant Research Center Ahvaz Jundishapur University of Medical Sciences Ahvaz Iran; ^3^ Nanotechnology Research Center Ahvaz Jundishapur University of Medical Sciences Ahvaz Iran; ^4^ Student Research Committee Ahvaz Jundishapur University of Medical Sciences Ahvaz Iran

**Keywords:** allergic contact dermatitis, skin effects, topical treatment

## Abstract

**Background:**

Doxepin (DX) is used orally to relieve itching but can cause side effects like blurred vision, dry mouth, and drowsiness due to its antimuscarinic effect. To reduce these adverse effects and improve skin permeation, DX is being developed in topical formulations. This study aims to improve DX skin absorption by developing a microemulsion (ME) formulation (ME‐DX).

**Method and Materials:**

ME formulations containing 5% DX were prepared using the phase diagram method with a mixture of essential oil (oleic acid), surfactant (Tween 80 and Labrazol), and cosurfactant (propylene glycol or PG). The physicochemical properties of these formulations were assessed to improve the topical application of DX.

**Results:**

ME droplet sizes ranged from 9.8 to 61.6 nm, with viscosities between 114 and 239 cps. Studies showed a significant correlation between DX release percentages and viscosity. ME‐DX‐2 released 46.51% of the drug after 24 h. Selected ME‐DX formulations demonstrated Weibull, Log Wagner, and zero‐order release kinetics, and improved skin permeability. ME‐DX‐8 exhibited an eightfold increase in *J*ss and *P* parameters compared to the control group. MEs maintained 99.9% of DX after 6 months without color changes or phase separation, indicating long‐term stability.

**Conclusion:**

This research demonstrates that altering the content and composition of MEs can change the physicochemical properties and permeability characteristics of DX when introduced into rats. Additionally, ME formulation shows promise as an effective vehicle for topical DX delivery in atopic dermatitis treatment.

## 1 Introduction

Microemulsions (MEs) are mixtures of oil and water stabilized by surfactants and cosurfactants. These mixtures remain stable at a thermodynamic level and flow easily due to their low viscosity [1, 2]. MEs fall into three main types: oil‐in‐water, water‐in‐oil, and bicontinuous phase, each with distinct characteristics and uses [3]. When used for topical and transdermal drug delivery, MEs offer several benefits. They can hold large amounts of drugs, increasing the concentration difference between the ME and the skin. This promotes drug movement into the skin. Additionally, ME components work together to improve drug penetration through the skin, improving overall delivery. The water phase, oil phase, and surfactant–cosurfactant combinations all contribute to increasing drug flux, making MEs effective for transdermal delivery [4, 5, 6].

Doxepin (DX), a tricyclic antidepressant compound (TAC), strongly blocks H1 and H2 receptors. It outperforms other common TACs like Diphenhydramine and Hydroxyzine as an H1 blocker, showing 775 times and 56 times greater potency, respectively [7, 8]. DX also blocks muscarinic acetylcholine receptors, which can cause side effects such as blurry vision, dry mouth, and difficulty urinating. Given these oral side effects, researchers explored topical application as an alternative [9]. Notably, topical DX significantly raised the threshold for histamine‐induced itching, suggesting its potential for treating itch‐related conditions [10].

Against this background, this study aimed to develop DX formulations, evaluate their physical and chemical properties, and examine how well they penetrate the skin using an ex vivo model.

## 2 Material and Method

### 2.1 Material

We obtained DX powder from Ramofarmin Company in Iran. Other ingredients, including oleic acid, Labrasol, Tween 80, and PG, were sourced from Merck (Germany). All chemicals and solvents used were of analytical grade. We used only fresh double‐distilled water in our experiments to ensure accuracy.

### 2.2 Animal

Our study used male adult Wistar rats weighing between 200 g and 250 g. We conducted this research with approval from the Animal Ethical Committee at Ahvaz Jundishapur University of Medical Sciences (permit number IR.AJUMF.REC.1395.131).

### 2.3 DX Assay

We measured DX using a UV spectrophotometer (Biowave‐2, Biochrom, England) at 292 nm in a phosphate buffer solution (PBS) with a pH of 7.4.

### 2.4 DX Solubility

We selected oleic acid as the oil, Tween 80 and Labrasol as surfactants, and PG as the cosurfactant. We then evaluated DX's solubility in these components. We placed 5 mL of each substance in a beaker and added DX. The mixtures were stirred for 48 h at 25°C, then centrifuged for 15 min at 3000 rpm. We collected the supernatant and sediment and then measured the concentration of the dissolved drug at 292 nm using UV spectrophotometry.

### 2.5 Pseudo‐Ternary Phase Diagram Construction

To examine the concentration range of components within ME spots, we used the water titration method to create pseudoternary phase diagrams [11]. We prepared two phase diagrams with weight ratios of 2:1 and 4:1 for (Labrasol—Tween 80/PG). We then mixed the oil phase (Oleic acid) with the surfactant/cosurfactant mixture at weight ratios from 1:9 to 9:1. These mixtures were gradually diluted with double distilled water under moderate agitation. Samples that turned into clear liquids were identified as ME.

### 2.6 Preparation of DX MEs

We used a full factorial design to create eight ME‐DX formulations. This method involved adjusting three key variables at two levels: oil percentage (%oil), water percentage (%W), and surfactant/cosurfactant ratio (S/C). We prepared the eight formulations using different levels of oil (5% and 20%), water (20% and 30%), and S/C mixing ratio (2:1 and 4:1). We chose the ME formulations based on the pseudoternary phase diagram, using weight ratios of 2:1 and 4:1 for Tween 80‐Labrasol/PG, as shown in Table 1.

**TABLE 1:** Composition of selected Doxepin microemulsion formulations.

Formulation	Factorial	Surfactant/cosurfactant	% Oil	% Surfactant + cosurfactant	% Water	% Drug
ME‐DX‐1	+++	4:1	20	45	30	5
ME‐DX‐2	++−	4:1	20	55	20	5
ME‐DX‐3	+−+	4:1	5	60	30	5
ME‐DX‐4	+−−	4:1	5	70	20	5
ME‐DX‐5	−−−	2:1	5	60	30	5
ME‐DX‐6	−−+	2:1	5	70	20	5
ME‐DX‐7	−+−	2:1	20	55	20	5
ME‐DX‐8	−++	2:1	20	45	30	5

To prepare the formulations, we first added DX (5%) to the oil phase. We then slowly incorporated the S/C mixture and a specific amount of double‐distilled water, stirring continuously at room temperature until we achieved a uniform mixture.

### 2.7 Droplet Size Measurements

We measured the droplet size of the ME samples at 25°C ± 1°C using a particle size analyzer (SCATTER SCOPE 1 QUIDIX, South Korea).

### 2.8 Viscosity Measurements

We assessed the viscosity of the ME samples using a Brookfield viscometer (DV‐II + Pro Brookfield, USA) with spindle number 34 at 25°C ± 0.5°C.

### 2.9 Differential Scanning Calorimetry (DSC)

We performed DSC assessments using a Mettler Toledo DSC1 star system with a refrigerated cooling system. We placed 5–10 mg of each ME sample in hermetically sealed aluminum pans to prevent water evaporation, using an empty hermetically sealed pan as the reference. We tested the samples across a temperature range from +30°C to −50°C at a scan rate of 10°C/min. We determined enthalpy changes (ΔH) from the endothermic and exothermic peaks observed in the DSC thermograms [12].

### 2.10 Stability Studies

We evaluated the physical stability of each ME formulation through centrifuge stress tests and temperature stability assessments. We stored the MEs under various temperature conditions (4°C, 25°C, 37°C, and 75% ± 5% relative humidity) following ICH guidelines for 6 months. After this period, we analyzed the formulations to measure changes in physicochemical characteristics, including clarity, phase separation, viscosity, and particle size in relation to time and temperature.

Additionally, we processed the MEs using a High‐Speed Brushless Centrifuge (MPV‐350R, POLAND) at 10000 rpm for 30 min at room temperature. After centrifugation, we visually assessed phase separation to check the physical stability of the formulations [13, 14].

### 2.11 Rat Skin Sample Preparation

Wistar rats weighing 200–250 g were used for skin permeation tests. The animals were euthanized with a ketamine/xylazine mixture. After shaving the abdominal area, the skin was removed and its inner surfaces were treated with cold acetone. The samples were then frozen at −20°C. For permeation tests, the skin samples were thawed, cut into pieces, and placed on diffusion cells with the epidermis facing the donor side.

### 2.12 Drug Release Study

Franz diffusion cells with a 4.906 cm^2^ specific diffusion area were used to assess drug release from ME‐DX formulations [15]. A cellulose membrane, hydrated in distilled water for 24 h, was placed between donor and receptor compartments before each experiment. Then, 3 g ME‐DX samples were added to the release system. The cells were filled with PBS buffer (pH 7.4) and stirred at 200 rpm. At set intervals (0.5, 1, 2, 3, 4, 5, 6, 7, 8, and 24 h), 2 mL ME‐DX samples were withdrawn, analyzed using a UV spectrophotometer, and replaced with fresh PBS. The cumulative quantity of released DX was plotted against time and analyzed using various kinetic models including Log Wagner, Weibull, Higuchi, zero order, and first order. The highest *r*2 value indicated the most likely release mechanism [16].

### 2.13 Permeability Experiments

In vitro permeation was evaluated using a custom vertical diffusion cell with an effective diffusion area of approximately 4.906 cm^2^. The receptor compartment contained 35 mL PBS (pH 7.4). Whole skin samples, hydrated before use, were fixed between the donor and receptor phases of the Franz cell, with the stratum corneum (SC) layer of rat skin facing the donor phase. The donor phase was filled with 5 g of each ME‐DX sample. Diffusion cells were placed on a heater stirrer at 37°C ± 0.5°C, and the receptor phase was continuously stirred at 200 rpm using small magnetic bars. At specific intervals (0.5, 1, 2, 3, 4, 5, 6, 7, 8, 24, 32, and 48 h), 2 mL samples were removed from the receptor medium and immediately replaced with 2 mL PBS to maintain sink conditions. A UV spectrophotometer detected the amount of permeated DX in the samples at 292 nm. Free drug MEs and 5% DX/water solution served as negative and positive controls, respectively [17].

### 2.14 Data Analysis and Statistics

The cumulative permeated DX per unit area was calculated and plotted against time. The steady‐state flux (*J*ss, mg/cm^2^ h) was calculated from the linear portion of the permeation curve. The permeability coefficient (*P*, cm/h) for DX through the skin was determined using the following equation:

$$P=\frac{J\mathrm{ss}}{Cv}$$

where *J*ss is the steady‐state flux and *C*v is the DX concentration in the donor medium [4, 12].

To compare the permeability parameters of the ME‐DX samples to the control (5% DX water solution), the enhancement ratio (ER) was calculated as:

$$\mathrm{ER}=\frac{\text{Permeability parameter amount of}\;\mathrm{ME}\;\text{formulation}\;}{\text{Permeability parameter amount of control}}$$

All experiments were conducted in triplicate, with results reported as mean ± Standard deviation (SD). Statistical analysis was performed using one‐way analysis of variance (ANOVA), with significance set at *p* < 0.05.

## 3 Results

### 3.1 Solubility Study

The solubility of DX in various excipients was evaluated. Table 2 presents the solubility results of DX. Among the tested compounds, DX showed the highest solubility in oleic acid.

**TABLE 2:** Solubility of doxepin in oil, surfactant, and cosurfactant (*n* = 3, Mean ± SD).

Phase type	Excipient	Solubility(mg/mL)
Oil	Oleic acid	14.1 ± 0/02
Surfactant	Tween80	8.3 ± 0/05
Surfactant	Labrasol	7.1 ± 0.1
Cosurfactant	PG	9.8 ± 0/2

### 3.2 Phase Diagram

**FIGURE 1:**
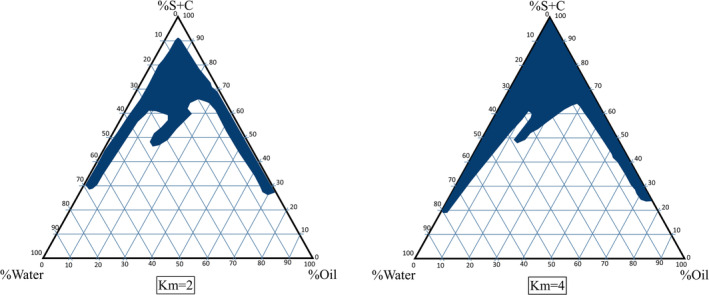
Pseudo‐ternary phase diagrams of ME formation at km = 2 and km = 4 ratios of Labrasol‐Tween 80/PG.

The phase diagram system consisted of oleic acid (oil phase), Tween 80‐Labrasol (surfactant), and PG (cosurfactant). These components were chosen based on their drug solubility capacity and ability to form MEs. The phase diagram helped identify ME zones. Two‐phase diagrams were created at surfactant/cosurfactant ratios of 2/1 and 4/1, as shown in Figure 1. These diagrams reveal that a higher surfactant/cosurfactant ratio expands the ME region and increases water content within the ME structure. Eight formulations were selected based on factorial design for in vitro skin permeability testing.

### 3.3 Characterization of DX MEs

The viscosity, droplet size, polydispersity index (PDI), and pH of the DX ME formulations were examined, with results presented in Table 3. ME formulations showed average viscosity values between 114 and 239 cps, pH levels from 5.2 to 6.2, and droplet sizes ranging from 9.8 to 61.6 nm. ANOVA analysis revealed a significant correlation between ME droplet sizes and the independent variables. As the surfactant to cosurfactant ratio of ME‐DX increased, so did the droplet size. This suggests that smaller droplet sizes are linked to a larger surface area, potentially improving bioavailability and skin permeation [18].

**TABLE 3:** Physicochemical properties of selected Doxepin microemulsions (*n* = 3, Mean ± SD).

Formulation	pH	Viscosity (cps)	PDI	Droplet size (nm)
ME‐DX‐1	5/2 ± 0/1	144 ± 2/6	0/401 ± 0.002	61/1 ± 1/6
ME‐DX‐2	5/2 ± 0/1	151 ± 3/2	0/408 ± 0.003	37/2 ± 1/2
ME‐DX‐3	5/3 ± 0/1	222 ± 4/1	0/405 ± 0.011	17/1 ± 1/4
ME‐DX‐4	5/3 ± 0/2	239 ± 5/3	0/411 ± 0.012	15/6 ± 0/9
ME‐DX‐5	5/4 ± 0/3	132 ± 2/2	0/408 ± 0.002	9/8 ± 0/1
ME‐DX‐6	5/9 ± 0/2	193 ± 3/1	0/412 ± 0.012	19/8 ± 2/4
ME‐DX‐7	6/1 ± 0/1	124 ± 1/2	0/405 ± 0.005	29/1 ± 1/8
ME‐DX‐8	6/1 ± 0/2	114 ± 1/3	0/404 ± 0.004	12/1 ± 1/2

The PI values, all below 0.5, indicate a narrow droplet size distribution within the ME formulations. For comparison, Tavakli et al. reported a pH of 5.2 for their DX cream formulation [19], while Sandig et al. noted a pH of 6.7 for their DX nanoemulsion [20]. The pH values measured in this study fall within a range considered suitable for topical application.

### 3.4 DX Release Evaluation From ME Formulation

**FIGURE 2:**
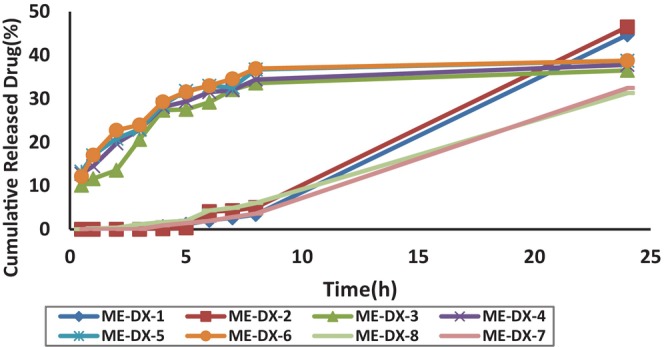
In vitro release profile of Doxepin from microemulsion formulations.

Figure 2 shows the release profile of DX from selected DX‐MEs. The analysis indicates that 46.5% of DX was released within 24 h for ME‐DX‐2. Table 4 summarizes the DX release percentages and release kinetics for the ME formulations.

**TABLE 4:** Release percentage and kinetics of selected Doxepin microemulsion formulations (*n* = 3, Mean ± SD).

Formulation	Kinetic of release	*R* ^2^	% R2 h	% R24 h
ME DX 1	Weibull	0/9978	Not Release	44/66 ± 0/38
ME DX 2	Zero	0/9915	Not Release	46/51 ± 1/42
ME DX 3	Log Wagner	0/9025	13/57 ± 0/05	36/51 ± 0/31
ME DX 4	Log Wagner	0/9492	19/73 ± 0/43	37/80 ± 0/19
ME DX 5	Log Wagner	0/9402	20/84 ± 0/04	38/72 ± 0/15
ME DX 6	Log Wagner	0/9406	22/76 ± 0/06	38/74 ± 0/10
ME DX 7	Zero	0/9808	Not Release	32/45 ± 0/16
ME DX 8	Zero	0/9796	0/34 ± 0/06	31/29 ± 0/26

ANOVA analysis revealed significant correlations between:

1. DX released in 2 h (R2 h) and independent variables

- %Oil
- Surfactant/cosurfactant ratio (*p* < 0.05) in DX‐ME formulations

2 DX released in 24 h (R24 h) and the independent variable %oil (*p* < 0.05)

These findings suggest that increasing the oil percentage in the phase leads to higher R24 h in ME samples. Previous research has shown that smaller droplet sizes result in faster drug molecule release [21].

### 3.5 Differential Scanning Calorimetry Studies

**FIGURE 3:**
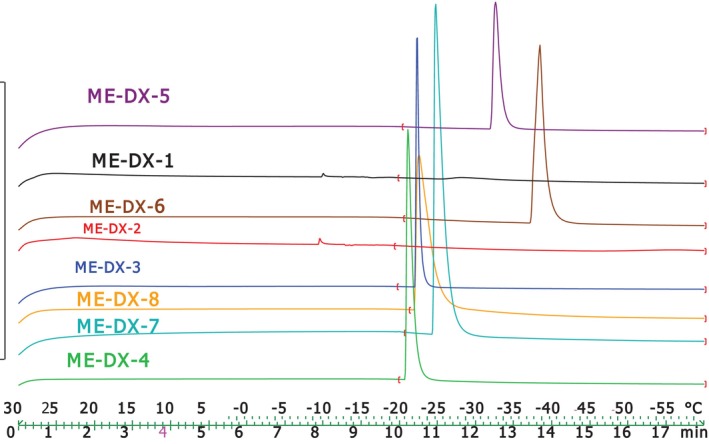
DSC cooling thermograms of ME‐DX formulations.

The DSC cooling thermograms of ME‐DX formulations, shown in Figure 3, provide information about transition temperature and enthalpy values, as recorded in Table 5. The cooling curves of the ME formulation reveal distinct temperature points: bulk water (free water) at 0°C and bound water between −9°C and −38°C.

**TABLE 5:** Thermal parameters of selected Doxepin microemulsion formulations (*n* = 3, Mean ± SD).

Formulation	TM_1_ (°C)	TM_**2**_ (°C)	ΔH_1_ (mJ/mg)	ΔH_2_ (mJ/mg)
	0	−10 ± 0.01	0.8155 ± 0.001	2.9237 ± 0.3
ME DX 2	0	−9 ± 0.001	1.3155 ± 0.1	−0.3143 ± 0.01
ME DX 3	0	−22 ± 0.3	0	78.4456 ± 3.2
ME DX 4	0	−21 ± 0.2	0	160.5 ± 1.2
ME DX 5	0	−32 ± 0.4	0	73.2273 ± 1.5
ME DX 6	0	−38 ± 0.5	0	195.4 ± 1.9
ME DX 7	0	−24 ± 0.3	0	309.4 ± 3.6
ME DX 8	0	−22 ± 0.1	0	239.8 ± 2.3

ANOVA analysis shows a significant correlation (*p* < 0.05) between the bound melting transition temperature (Tm2) and the independent variables, including %oil and surfactant/cosurfactant ratio. Notably, enthalpy increases as the surfactant/cosurfactant ratio rises and the oil phase percentage decreases.

This understanding of water's thermal behavior offers valuable insight into the microstructure of MEs [22]. Our results align with the conclusions proposed by Podlogar et al. [18].

### 3.6 Stability Study

This study showed that all ME formulations remained homogeneous and stable for 6 months. The ME‐DX systems maintained physical and chemical stability throughout this period, with no phase separation, flocculation, or coalescence observed under various temperature conditions and during centrifugation. These results point to the strong thermodynamic stability of the formulated DX system.

The selected MEs retained 99.9% of their DX content after 6 months of storage. They remained visually unchanged, with no color shifts or phase separation. This suggests that ME‐DX formulations can effectively preserve the drug for extended periods without the need for antioxidants.

### 3.7 Ex Vivo Skin Permeation Study

The permeability parameters of different ME‐DX formulations are shown in Table 6. Significant correlations were found between *J*ss and the surfactant/cosurfactant ratio (*p* < 0.05), with higher ratios leading to increased *J*ss values in ME‐DX formulations. For instance, the *J*ss of DX for ME‐DX‐8 was 0.0520 mg/cm^2^ h, which is nine times higher than the control (5% DX solution).

**TABLE 6:** In vitro permeability parameters of Doxepin microemulsion formulations through rat skin (*n* = 3, Mean ± SD).

Formulation	*J*ss, mg/cm^2^ h	T_**Lag**_, h	D_**app**_, cm^2^/h	P_**app**_ (cm/h)	ER_**flux**_	ER_**D**_	ER_**P**_
Control	0.0059 ± 0.0001	7.39 ± 0.05	0.0058 ± 0.0002	0.0001 ± 0.0001	—	—	—
ME‐DX‐1	0.0215 ± 0.0062	2.92 ± 0.2	0.0267 ± 0.0209	0.0004 ± 0.0001	3.64 ± 0.05	4.55 ± 0.56	3.64 ± 0.05
ME‐DX‐2	0.0324 ± 0.0044	1.11 ± 0.08	0.0938 ± 0.0917	0.0006 ± 0.00089	5.5 ± 0.75	15.98 ± 0.64	5.5 ± 0.75
ME‐DX‐3	0.0176 ± 0.0005	0.65 ± 0.2	0.0878 ± 0.0288	0.0004 ± 0.0001	2.98 ± 0.09	14.98 ± 1.91	3.79 ± 0.24
ME‐DX‐4	0.0186 ± 0,0118	2.56 ± 0.9	0,0622 ± 0.0715	0.0004 ± 0.0002	3.16 ± 0.01	10.60 ± 0.19	3.16 ± 0.01
ME‐DX‐5	0.0109 ± 0.0037	3.48 ± 0.2	0.0194 ± 0.0132	0.0002 ± 0.00075	1.85 ± 0.63	3.30 ± 0.09	1.85 ± 0.63
ME‐DX‐6	0.0099 ± 0.0002	3.45 ± 0.5	0.0159 ± 0.0027	0.0002 ± 0.0004	1.68 ± 0.03	2.71 ± 0.4596	1.68 ± 0.03
ME‐DX‐7	0.0099 ± 0.0028	4.99 ± 0.06	0.0413 ± 0.0501	0.0003 ± 0.0084	1.67 ± 0.47	7.03 ± 1.54	2.52 ± 0.71
ME‐DX‐8	0.0520 ± 0.0040	4.79 ± 0.4	0.0113 ± 0.0011	0.0010 ± 0.0080	8.82 ± 0.68	1.93 ± 0.18	8.82 ± 0.68

No significant correlations (*p* > 0.05) were observed between Dapp and Tlag with the independent variables. However, Dapp values in ME formulations were consistently higher than in the control. The maximum Tlag of DX for ME‐DX‐7 was 4.99 h, with all ME formulations showing lower Tlag levels than the control sample.

The permeability coefficient (*P*) showed a significant correlation with the surfactant/cosurfactant ratio. Higher ratios resulted in higher *P* values. For example, the *P* of DX for ME‐DX‐8 was 0.0010 cm/h, 10 times higher than the control.

ERflux also correlated significantly with the surfactant/cosurfactant ratio, with increases in the ratio leading to higher ERflux values in ME‐DX formulations. All ME‐DX formulations, regardless of their composition, markedly increased flux and permeability coefficient from rat skin. These findings align with previous studies highlighting the benefits of MEs as enhancers in dermal delivery [23, 24, 25, 26].

The composition of the surfactant/cosurfactant mixture in ME samples clearly influenced the skin permeation rate of DX. This study demonstrates the potential of ME formulations to improve the dermal delivery of DX.

## 4 Discussion

The oral administration of DX is associated with side effects, prompting interest in developing topical drug delivery systems [27, 28]. The skin's primary defense mechanism, particularly the stratum corneum (SC), restricts drug molecule movement. MEs have emerged as promising vehicles for topical drug delivery, offering several advantages.

MEs have a high solubilizing capacity, allowing for significant drug incorporation. They can also improve the steady‐state flux of drugs by modifying the drug's affinity to the internal phase, favoring partitioning into the SC. This can be achieved by using different internal phases, altering their proportion in the ME, or adjusting their properties [29].

Drugs can penetrate the skin through two main routes: intercellular and transcellular. The intercellular route, involving neutral lipids in bilayers, is the primary pathway for most lipophilic drugs [15, 30]. ME formulations can permeate the SC layer while maintaining structural integrity, affecting both polar and lipid pathways.

The lipophilic portion of the ME interacts with the SC through various mechanisms. The drug dissolved in the ME's lipid component may directly permeate into the SC lipids, or the lipid vesicles may intercalate between the SC lipid chains, disrupting its bilayer structure. These interactions likely increase the lipid pathway's permeability to DX.

The ME's hydrophilic domain hydrates the SC, playing a crucial role in drug permeation. As the ME's aqueous phase enters the polar pathway, it increases the interlamellar volume of SC lipid bilayers, disrupting their interfacial structure [29, 31, 32]. The main approach involves assessing the skin penetrability of drug molecules by determining the maximum steady‐state flux (*J*ss) across the skin. Results presented in Table 6 from ex vivo permeability studies confirm ME‐DX's superiority over the DX solution as a candidate for topical delivery.

Research on various compounds as enhancers for transdermal ME formulations, including fatty acids, alcohols, sulfoxides, surfactants, and amides, found that enhancers with an unsaturated C18 alkyl chain, particularly oleic acid, performed best. This is due to fatty acids' ability to permeate the skin bilayer, creating permeable pathways [33, 34]. Oleic acid, a popular enhancer, significantly increased the flux of ME formulations through the skin membrane [35].

Propylene glycol (PG) improves medicine permeability similarly to ethanol, boosting the permeability of ME systems [36, 37]. Nonionic surfactants are often used in topical formulations as solubilizing agents. In the ME formulations studied, oleic acid, PG, and water act as permeation enhancers through various mechanisms, including disrupting the organized intercellular lipid structure of the stratum corneum (SC) and fluidizing SC lipids [38, 39, 40].

Based on this study's data and analysis, the ME‐DX formulations developed show promise for dermal application. However, further in vivo efficacy studies are needed to fully validate and demonstrate their potential as an effective dermal delivery system for DX. These additional studies will provide a more comprehensive understanding of the formulations' performance in real‐world conditions and their therapeutic efficacy.

## 5 Conclusion

This study demonstrates that the optimal ratios of water, oil, surfactant, and cosurfactant in the ME formulation significantly affect its physicochemical properties and permeability parameters. The DX release kinetics in all tested MEs followed various models, including Log Wagner, Weibull, and zero‐order models, resulting in controlled release patterns compared to the DX solution. The ME system notably improved DX stability. Moreover, the investigated MEs showed a marked increase in both permeation rate and permeability coefficient through rat skin samples. These findings suggest that the ME formulations have potential applications in transdermal drug delivery systems.

## Author Contributions

A.S., N.J., and Ar.S. conceptualized and designed the evaluation and wrote the initial manuscript draft. A.S. and E.M. contributed to the evaluation design, conducted part of the statistical analysis, and assisted in manuscript preparation. A.S. and H.H.K. jointly reassessed the data, refined the statistical analysis, and revised the manuscript. H.D. gathered and interpreted the clinical data, and contributed to manuscript revisions. All authors (H.H.K., E.M., H.D., N.J., Ar.S., and A.S.) gave final approval for publication.

## Ethics Statement

We conducted this research with approval from the Animal Ethical Committee at Ahvaz Jundishapur University of Medical Sciences (permit number IR.AJUMF.REC.1395.131).

## Conflicts of Interest

The authors declare no conflicts of interest.

## Data Availability

The data that support the findings of this study are available on request from the corresponding author. The data are not publicly available due to privacy or ethical restrictions.
